# Two Isomeric Thienoacenes in Thin Films: Unveiling the Influence of Molecular Structure and Intermolecular Packing on Electronic Properties

**DOI:** 10.1021/acs.jpcc.4c06741

**Published:** 2024-12-02

**Authors:** Christos Gatsios, Maximilian Dreher, Patrick Amsalem, Andreas Opitz, Remy Jouclas, Yves Geerts, Gregor Witte, Norbert Koch

**Affiliations:** † Institut für Physik & Center for the Science of Materials Berlin (CSMB), Humboldt-Universität zu Berlin, Berlin 12489, Germany; ‡ Department of Physics, Philipps-Universität Marburg, Marburg 35037, Germany; § Laboratoire de Chimie des Polymères, Faculté des Sciences, Université Libre de Bruxelles (ULB), Boulevard du Triomphe, CP 206/01, Bruxelles 1050, Belgium; ∥ International Solvay Institutes for Physics and Chemistry, Université Libre de Bruxelles (ULB), Boulevard du Triomphe, CP 231, Bruxelles 1050, Belgium; ⊥ Helmholtz-Zentrum Berlin für Materialien und Energie GmbH, Berlin 12489, Germany

## Abstract

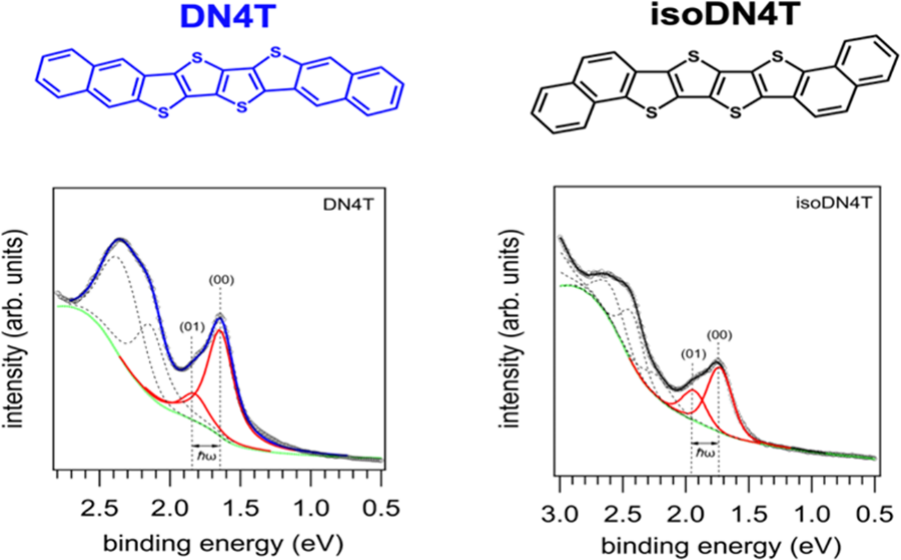

Isomerism of molecular structures is often encountered in the field of organic semiconductors, but little is known about how it can impact electronic and charge transport properties in thin films. This study reveals the molecular orientation, electronic structure, and intermolecular interactions of two isomeric thienoacenes (DN4T and isoDN4T) in thin films, in relation to their charge transport properties. Utilizing scanning tunneling microscopy (STM), angle-resolved photoemission spectroscopy (ARUPS), and near-edge X-ray absorption fine structure measurements (NEXAFS), we systematically analyze the behavior of these isomers from submonolayer to multilayer coverage on highly ordered pyrolytic graphite (HOPG) as substrates. We find that at submonolayer coverage both DN4T and isoDN4T molecules predominantly adopt a nearly flat-lying orientation on the surface, minimizing intermolecular interactions. The distinct emission features of the highest occupied molecular orbital (HOMO) level in ARUPS enables the determination of molecular reorganization energies. These are found to be in good agreement with theoretical predictions, suggesting superior charge transport in DN4T compared to isoDN4T. Notably, thickness-dependent photoemission measurements reveal a significant splitting (approximately 450 meV) of the HOMO level of isoDN4T, attributed to polarization-induced effects rather than wave function overlap, indicating a nuanced interplay between molecular packing and electronic properties. Our results underscore the importance of molecular packing and substrate interactions in determining the electronic structure and transport properties of organic semiconductor thin films. Substrate-induced polymorphism and the crucial role of polarization-induced effects influencing charge transport are highlighted. These insights are pivotal for future engineering of molecular and thin film structures, aiming to enhance the performance of organic semiconductor-based devices.

## Introduction

Knowledge of the intricate interplay between molecular structure and packing in organic semiconductors is pivotal for the development of high-performance thin film electronic and optoelectronic devices. These structural characteristics profoundly influence the electronic energy level landscape, dictating the behavior of charge carriers within the material. Several studies have shown that intramolecular and intermolecular vibrations in organic semiconductors can significantly couple with charge carriers during transport, potentially leading to kinetic energy loss and increased charge localization.^1−8^ This phenomenon can be quantitatively described within the Holstein model, which defines the polaron binding energy, or the related quantity of reorganization energy, both representing the electronic energy required by the molecular system to accommodate the excess charge.^2,9,10^ Furthermore, the molecular structure and packing critically determine the ionization energy and electron affinity, which are essential parameters for assessing electrochemical stability and charge injection efficiency.^11−13^

**Figure 1:**
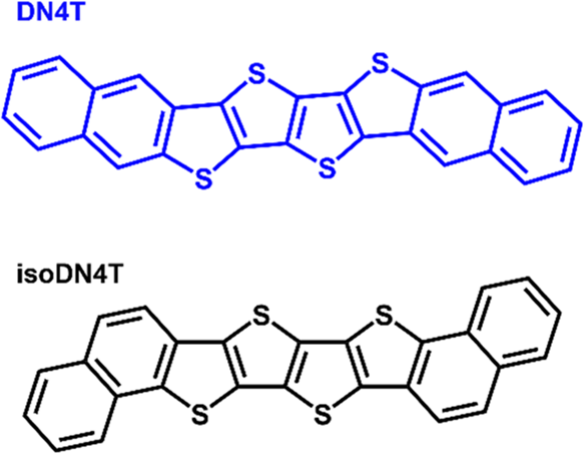
Molecular structures of DN4T and isoDN4T.

Given the key importance of these structure–property relationships, our present work aims to elucidate the impact of molecular structure and packing on the electronic and charge transport properties of organic semiconductor thin films of two isomers, DN4T (naphtho[2,3-*b*]thieno-[2‴,3‴:4″,5″]thieno[2″,3″:4′,5′]thieno[3′,2′-*b*]naphtho[2,3-*b*]thiophene) and isoDN4T (naphtho[1,2-*b*]thieno-[2‴,3‴:4″,5″]thieno[2″,3″:4′,5′]thieno[3′,2′-*b*]naphtho[1,2-*b*]thiophene) (for molecular structures, see Figure 1), which, despite their similar molecular structures, exhibit markedly different transport behaviors. Previous investigations by Jouclas et al. have demonstrated a significantly higher hole carrier mobility in DN4T compared to isoDN4T, a disparity attributed to a smaller reorganization energy and a larger transfer integral of DN4T.^14^

While the previous work relied on theoretical calculations to infer the reorganization energy and transfer integral from single molecules and single crystals, our present work explores these quantities from an experimental standpoint on DN4T and isoDN4T thin films. We performed ultraviolet photoemission spectroscopy (UPS) and angle resolved photoemission (ARUPS) measurements on submonolayer to multilayer coverage of DN4T and isoDN4T deposited on highly ordered pyrolytic graphite (HOPG). Earlier photoemission studies on planar conjugated molecules allowed to make a correlation between the molecular orientation and the observed angle-dependent photoelectron intensity of the highest occupied molecular orbital (HOMO) peak,^15−17^ which we exploit in the present work. However, we obtained quantitative information on the monolayer structure and molecular orientation from scanning tunneling microscopy (STM) and near-edge X-ray absorption fine structure (NEXAFS) measurements.

Although HOPG substrates are not typically used in organic semiconductor devices, they are employed here as they facilitate the growth of well-ordered molecular layers, as observed in structurally related molecules like pentacene and sexithiophene, which form ordered monolayers with a flat-lying molecular orientation.^18−21^ In this orientation, intermolecular interactions are minimized, and the electronic levels yield defined photoelectron peaks representative of individual molecules, allowing us to observe how intermolecular interactions evolve with increasing film thickness. Previous research has also shown that, while substrate choice can influence film morphology and molecular orientation, it does not significantly alter the fundamental packing pattern in organic thin films of this type.^22,23^

Our findings reveal a larger reorganization energy of isoDN4T compared to DN4T, as evidenced by the hole-vibration coupling observed in the UPS spectra. For multilayers of isoDN4T, a splitting of the HOMO-derived peak is observed, similar to previous observation for π-stacked lead-phthalocyanine bilayers.^24^ This was attributed to wave function overlap and interpreted to yield an approximation of the intermolecular transfer integral.^25^ Here, however, we favor the effect of intermolecular polarization-induced interactions to explain the splitting, which was shown previously to largely perturb the energy levels of the frontier orbitals.^26^

## Materials and Methods

The synthesis of DN4T and isoDN4T, whose molecular structures are depicted in Figure 1, followed the methodology outlined in previous work.^14^ Photoemission spectroscopy measurements were conducted using a helium discharge lamp, which emits unpolarized light with a photon energy of 21.21 eV. These experiments were performed at the ENERGIZE end-station of BESSY II, operated by the Helmholtz Zentrum Berlin für Materialien and Energie GmbH (HZB). The ENERGIZE setup comprises an ultrahigh vacuum (UHV) system, featuring a preparation chamber and an analysis chamber, both maintained at pressures around 10^–10^ mbar. The analysis chamber is equipped with a Scienta DA30 hemispherical analyzer. Pass energies of 5 and 2 eV were used to record the valence spectra and secondary electron cutoff, resulting in energy resolutions of 80 and 50 meV, respectively. Preparation and analysis of the thin films were conducted in situ. HOPG substrates were prepared by exfoliation with scotch tape under ambient conditions. Once introduced into the preparation chamber, the substrates were annealed at 450 °C for 1 h. Molecular evaporation onto the substrates was achieved in the preparation chamber, utilizing a quartz crystal microbalance to ensure a consistent molecular flux. The deposition rate for the films was maintained at approximately 0.1 Å/s.

The structure of DN4T and isoDN4T on HOPG was characterized using a scanning tunneling microscope (Omicron VT STM) under UHV conditions (approximately 10^–10^ mbar), employing etched tungsten tips. To minimize admolecular diffusion, data acquisition was performed at a temperature of approximately 110 K. Prior to film deposition, HOPG substrates (NT-MDT quality: A) were exfoliated under ambient conditions and subsequently annealed at 400 °C for 1 h under UHV conditions. The molecular films were deposited via sublimation from aluminum crucibles in resistively heated Knudsen cells, with deposition rates monitored by a quartz crystal microbalance, typically around 3 Å/min.

C 1s near-edge X-ray absorption fine structure (NEXAFS) measurements were carried out in partial electron yield (PEY) mode at the HE-SGM dipole beamline of BESSY II, which offers linearly polarized light (polarization factor: 0.91) and an energy resolution of approximately 300 meV at the C 1s edge. NEXAFS spectra were recorded at three different angles of incidence (30, 55 and 90°) to determine the average molecular orientation relative to the sample surface. The moderate photon flux of the dipole beamline allows to largely suppress radiation damages, that is confirmed by the observation of identical NEXAFS signatures at the beginning and end of dichroism measurements with different total exposure time. To minimize background signals from the π* resonances of the graphite substrate, monolayers of chemical vapor deposition (CVD) grown graphene on quartz glass (with graphene coverage >95%, Graphenea) were utilized as substrates, which were annealed to 330 °C before molecular film deposition. Studies indicate that, when transferred onto quartz, the graphene substrate yields molecular interactions comparable to those on HOPG, as it lacks the additional interactions that can occur on metal–supported graphene.^27,28^ Reference measurements from pristine graphene/quartz samples were conducted to subtract the residual background signal from the graphene layer.

We performed density functional theory calculations in ORCA for DN4T and isoDN4T single molecules to calculate the electron density of the highest occupied molecular orbitals (HOMO) of both molecules. For this we used the B3LYP functional and the 6-311G(d,p) basis set implemented by ORCA.^29^ The spatial distribution of the HOMO electron density was plotted in the Avogadro software.^30^ The analysis of the experimental data was done with custom scripts in Igor Pro 9 (Wavemetrics, Lake Oswego, OR).

## Results and Discussion

### Submonolayer Coverage: Monolayer Structure

**Figure 2:**
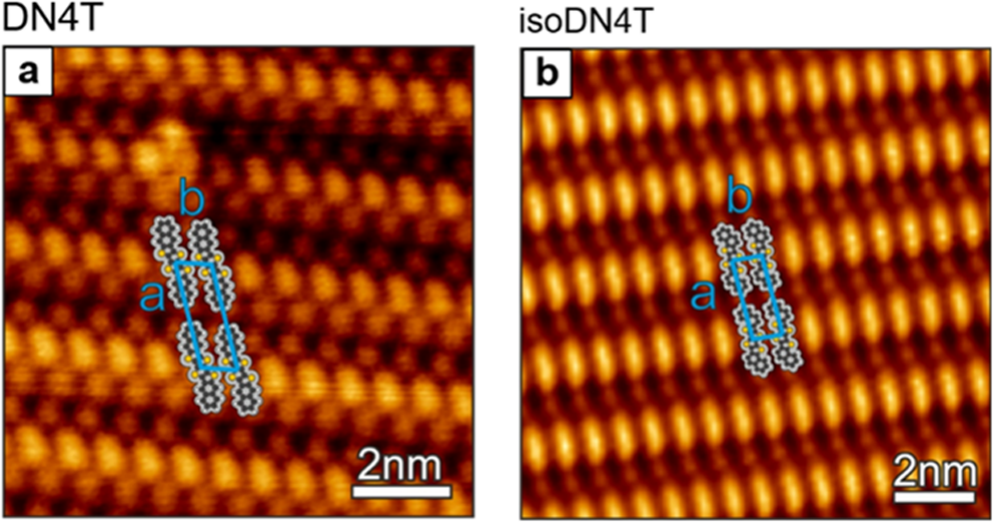
Scanning tunneling microscopy (STM) images showing the monolayer structure of (a) DN4T with unit cell parameters, *a* = 21.5 Å, *b* = 7.8 Å and φ = 68°, (b) and isoDN4T with ***a*** = 19.4 Å, ***b*** = 7.4 Å and φ = 84°, both on HOPG. (*U*_bias_ = 1.9 V, *I*_t_ = 120 pA).

Figure 2a,2b shows the STM images recorded for nominal molecular coverage of 4 Å, i.e., one monolayer (1 mL) of DN4T and isoDN4T on HOPG. In both cases, highly ordered lamellar motifs are observed, with the bright elongated structures corresponding to the aromatic backbones of DN4T and isoDN4T. Tentative modeling of the molecular arrangement as depicted in Figure 2a,2b suggests that the molecules are lying flat on the HOPG surface, consistently with previous reports for related molecules, such as pentacene and sexithiophene showing similar features.^18,21,31−33^

**Figure 3:**
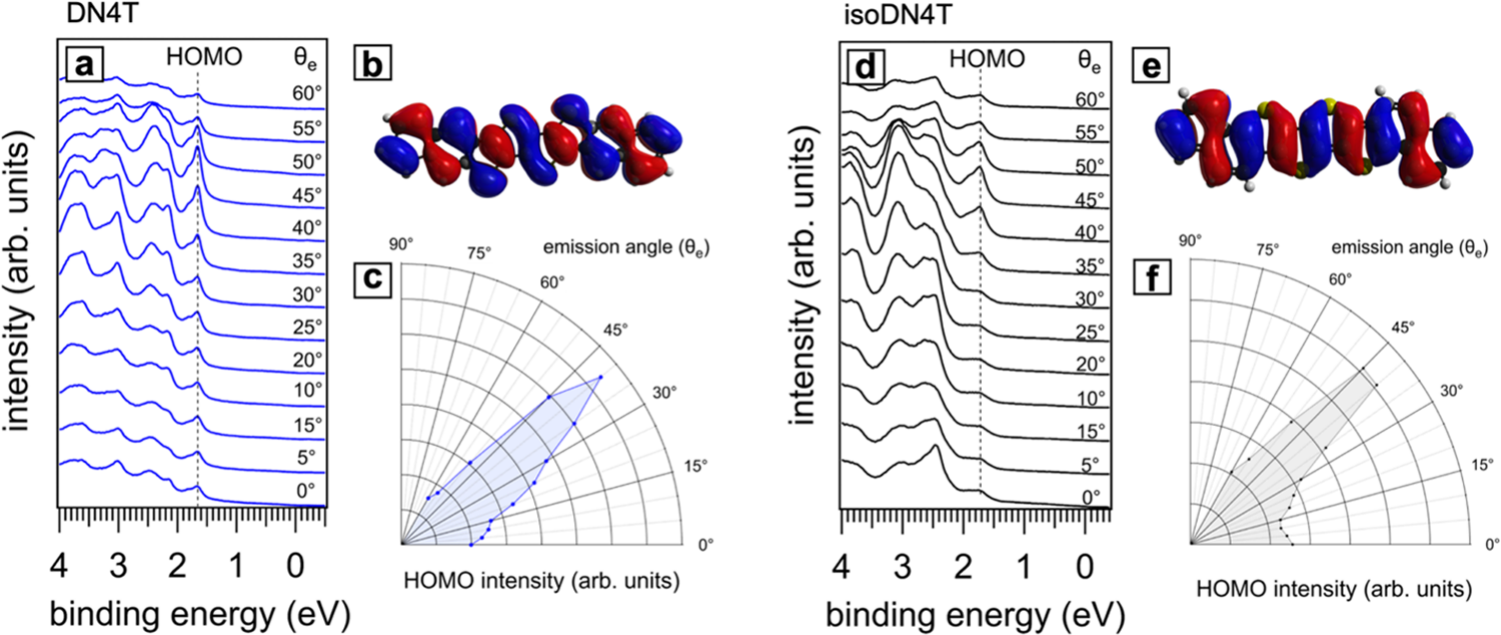
ARUPS spectra for monolayer (a) DN4T and (d) isoDN4T on HOPG. (c, f) Polar plots demonstrating the angular intensity distribution of the HOMO emission for DN4T and isoDN4T, respectively. θ_*e*_ denotes the photoelectron emission angle. The spatial distribution of the HOMO electron density obtained from calculations is shown in (b) for DN4T and (e) for isoDN4T, both characterized by nine orbital lobes.

X-ray photoelectron spectroscopy (XPS) measurements of the S 2p core level regions for DN4T and isoDN4T films on HOPG show no significant shifts in binding energy, indicating weak interactions between sulfur atoms and HOPG (Figure S1, Supporting Information). The S 2p_3/2_ binding energy (∼164.3 eV) aligns with values for similar molecules, confirming that the sulfur atoms remain bound within the molecular framework.^22^ Next, we performed angle-resolved UPS (ARUPS) to correlate the orientation of DN4T and isoDN4T molecules within monolayers on HOPG observed in STM with the photoemission spectra. This method is particularly effective in capturing the pronounced angular dependence of the HOMO peak intensity, a characteristic feature of flat-lying planar conjugated molecules.^15,16,34^ The ARUPS spectra, presented in Figure 3a for DN4T and Figure 3d for isoDN4T, reveal that, while the HOMO peaks do not exhibit energy dispersion, they show a significant intensity dependence with respect to the emission angle, as depicted in Figure 3c,3f for DN4T and isoDN4T, respectively. The HOMO peak, located at 1.65 eV for DN4T and at 1.72 eV for isoDN4T, reaches maximum intensity at emission angles of approximately 40° for DN4T and 45° for isoDN4T. To account for potential intensity variations resulting from changes in electron transmission to the analyzer during sample rotation, we normalized the spectral intensity at each emission angle based on angle-dependent measurements of the Fermi edge from a polycrystalline silver substrate (Figure S2, Supporting Information).

For an intuitive understanding of this observation, we approximate the HOMO wave function as that of an electron confined in a one-dimensional box, expressed as ϕ_HOMO_ ∼ sin(*k*_*x*_·*x*). In this way, we may define *k*_*x*_ along the molecular length as
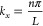
1

The molecular length, *L*, is derived from the unit cell parameters *a* obtained from the STM images. As depicted in Figure 3b,3e, for both molecules the HOMO is described by 9 lobes (i.e., *n* = 9). According to eq 1, *k*_DN4T_ = 1.32 Å^–1^ and *k*_isoDN4T_ = 1.45 Å^–1^. Next, the photoelectron intensity (*I*) is determined by the matrix element (*M*_if_), which, under the plane wave approximation for the final electron state, corresponds to the Fourier transform of the initial electron state (ϕ_HOMO_)^17,35,36^
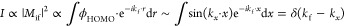
2

Eq 2 implies that the photoelectron intensity becomes appreciable only when the final momentum of the photoelectron matches the initial momentum of the electron at the HOMO molecular orbital. To support this argument quantitatively, we convert the emission angles corresponding to the highest HOMO intensity into wavenumbers using the following relation, found in standard photoemission textbooks,^37^
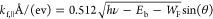
3

Here, *h*ν = 21.2 eV is the photon energy, *E*_b_ is the binding energy at the HOMO peak, *W*_F_ is the work function, θ is the emission angle between the sample surface normal and the analyzer, and *k*_f,||_ is the momentum component parallel to the surface, which is conserved upon electron transmission to the vacuum. Given eq 3, for DN4T we calculate 1.3 ± 0.2 Å^–1^ and for isoDN4T, 1.4 ± 0.2 Å^–1^, both values being in excellent agreement with our estimated values along the molecular length (see above). As expected, the observed momentum (*k*_f,||_) reflects mainly the *k_x_* component of the HOMO wave function, while the perpendicular in-plane component *k_y_* may be considered negligible due to the linear structure of the molecules. In addition, this good agreement provides further support for the molecules adopting a flat-lying orientation on the surface.

### Photoemission Vibrational Progression and Molecular Reorganization Energy

**Figure 4:**
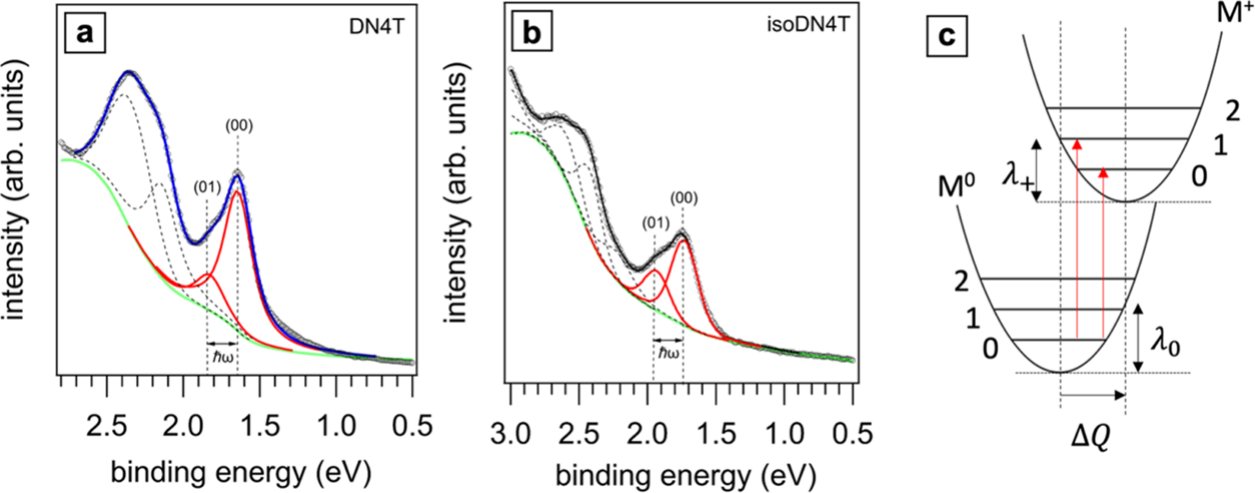
Deconvolution of the ARUPS spectra for (a) DN4T and (b) isoDN4T taken at 40° emission angle. The raw data are represented by empty circles. The red curves denote the emission from the HOMO, with the vertical dashed lines indicating the positions of the (00) and (01) vibronic peaks whose energy difference corresponds to the vibrational mode strongest coupled to the ionization. The green curve denotes the background, and the black dashed curves illustrate peaks from deeper energy levels. The least-squares fit results are shown in blue for DN4T and black for isoDN4T. Panel (c) provides a schematic of the involved transitions: The HOMO peak indicates an electronic transition from the neutral to the ionized state, creating a hole at the HOMO level. The electronic potential of the molecules as a function of generalized coordinates is approximated by a parabolic curve under the harmonic approximation. After ionization, the molecule may occupy any available vibrational state. The presence of a hole causes a geometry shift (Δ*Q*). The associated relaxation energies from neutral to ionized state and back are denoted as λ_0_ and λ_+_, respectively.

Figure 4a,4b shows zooms into the ARUPS spectra within the energy range close to the HOMO level. A close inspection of the high binding energy side of the principal HOMO-related peak (00) for both DN4T and isoDN4T unveils pronounced satellite peaks. These peaks are at 0.18 and 0.20 eV higher binding energy compared to the main HOMO peak of DN4T (at 1.65 eV binding energy) and isoDN4T (at 1.72 eV binding energy), respectively. The observed energy difference most likely accounts for the vibrational energy associated with the C–C stretching modes (typically in the 0.16–0.22 eV energy range) in the thiophene and benzene rings, a feature observed in photoemission and optical spectra of many conjugated organic molecules.^38,39^ In photoemission spectroscopy, the photoionization can bring a molecule to vibrationally excited states. The transition probability to each vibronic state, governed by the Franck–Condon factors, takes the form of a Poisson distribution, assuming the majority of the neutral molecules are in the ground vibrational state before ionization.^10^ This can be represented as given in eq 4.
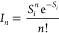
4

Here, *S_i_* corresponds to the Huang–Rhys factor for the *i*th vibrational mode, indicative of the strength of the hole-vibration coupling. This factor can be experimentally deduced, using eq 4, from the ratio of photoelectron intensities between successive vibrational peaks. Despite anticipating multiple vibrational modes to couple with the electronic transition,^14^ our experimental resolution is limited to ca. 110 meV, constraining our ability to discern lower energy vibrational modes, and molecular disorder also makes resolving more modes not possible in the present case.

The Huang–Rhys factors and vibrational energies derived from our experiments are, in turn, useful in calculating the polaron binding energy and reorganization energy, as has been demonstrated in a few prior photoemission studies on molecular systems.^40−43^ According to the Holstein model for small polarons,^9^ the polaron binding energy (*E*_pol_) quantifies the electronic energy cost associated with the geometrical relaxation of the molecule when a charge is present. Similarly, Marcus theory’s concept of reorganization energy (λ_reorg_) for intermolecular charge-transfer reactions,^44,45^ is the sum of the geometrical relaxation energies, λ_reorg_ = λ_0_ + λ_+_, during transition between the neutral ground state and the excited state, and vice versa, as illustrated in Figure 4c. The polaron binding energy is defined as half the reorganization energy. For rigid planar molecular structures, where the relaxation energies of the neutral and ionized state are typically identical,^3^ the reorganization energy can be expressed in terms of the vibrational energy (ℏω_vib_) and the Huang–Rhys factor
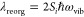
5

In our analysis, we deconvoluted the HOMO-related peaks of DN4T and isoDN4T by a single coupled vibrational mode, and we evaluated the reorganization energy using eq 5. Figure 4a,4b shows the peak fitting results, for the spectra obtained at 40° emission angle. The overall HOMO feature was fitted using two Voigt functions, with the Gaussian component accounting for instrumental broadening and the Lorentzian component representing the lifetime of the ionized molecular state (hole lifetime).^37^ To accurately determine the photoelectron intensity ratio *I*_(01)_/*I*_(00)_, we subtracted the background intensity, which includes inelastically scattered electrons (Shirley background^46,47^) and photoelectrons from the underlying graphite substrate.

The least-squares fitting results for isoDN4T yield a Gaussian width of 132 meV and a Lorentzian width of 108 meV, while DN4T returned values of 120 and 178 meV, respectively. This fitting procedure was applied to all angle-resolved spectra to explore any angular dependence of the Huang–Rhys factor and reorganization energy. As shown in Figures S3 and S4 (Supporting Information), we observed only a minor angular dependence. Although significant errors at larger emission angles due to lower photoelectron signals complicate the analysis, this effect may be analogous to that observed for pentacene on graphite. In that work, theoretical analyses attributed the effect to the coupling between molecular vibrational modes and optical phonons in graphite.^48^

Our angle-resolved analysis, restricted to a single vibrational mode, yields results that are consistent with previously reported theoretical values.^14^ As summarized in Table 1 and Figures S3–S5 (Supporting Information), our findings demonstrate that isoDN4T exhibits a larger reorganization energy and polaron binding energy compared to DN4T. This suggests that hopping transport is more efficient in DN4T than in isoDN4T, corroborating the findings.^14^ While these are within the confidence interval of our experimental results, the noted differences may hint at molecule–substrate interactions that were not considered in the theoretical calculations.^14^

**Table 1:** Experimental Parameters Determined by the Deconvolution of the HOMO Peak at 40° Emission Angle^a^

θ*_e_* = 40°	ℏω_vib_ (meV)	Huang-Rhys factor	λ_reorg,exp_ (meV)	*E*_pol_ (meV)	λ_reorg,theor_ (meV)
DN4T	186 ± 53	0.263 ± 0.003	98 ± 47	49 ± 24	152
IsoDN4T	201 ± 130	0.42 ± 0.03	168 ± 117	84 ± 59	213

a The vibrational energy ℏω_vib_ is obtained by the energy difference between (00) and (01) peaks. The Huang–Rhys factor by the intensity ratio *I*_(01)_/*I*_(00)_, the experimental reorganization energy (λ_reorg,exp_) from eq 5 and the polaron binding energy (*E*_pol_) as half the reorganization energy. The theoretical values for the reorganization (λ_reorg,theor_) are taken from ref (14). Available under a CC-BY 4.0 license. Copyright 2022. Jouclas et al.

### Molecular Multilayer Electronic Properties

**Figure 5:**
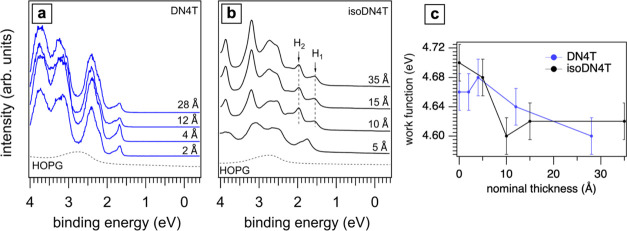
Thickness-dependent UPS spectra for DN4T (a) and isoDN4T (b). The dashed line represents the spectrum of the HOPG substrate. The spectra of isoDN4T show two new spectral features H_1_ and H_2_, appearing at a nominal thickness of 10 Å and persisting for higher coverage. Panel (c) shows the trend of the work function, for both DN4T and isoDN4T, upon increasing the nominal film thickness.

Figure 5a,5b illustrates the spectral evolution of DN4T and isoDN4T multilayers as revealed by thickness-dependent UPS measurements. For DN4T, the valence spectrum exhibits marginal changes with increasing coverage, with only a slight broadening likely due to increased structural disorder at higher thicknesses. In contrast, isoDN4T shows a significant spectral change from a nominal thickness of 10 Å on. Instead of one HOMO-derived feature below that thickness, two peaks, labeled H_1_ and H_2_ and separated by 420 meV, emerge in the HOMO spectral region. However, the peaks at higher binding energies, including the S 2p peak shown in Figure S1 (Supporting Information), are not apparently split and show minor changes with thickness. Beyond 10 Å nominal thickness, the spectra stabilize and showing no significant changes for higher thickness. In Figure 5c, the sample work function, as determined from the secondary electron cutoff region (Figure S6, Supporting Information), does not notably change within the experimental error range; the suggested trend toward slightly lower work function supports the notion of a push-back effect and physisorbed molecules on HOPG, as expected.

**Figure 6:**
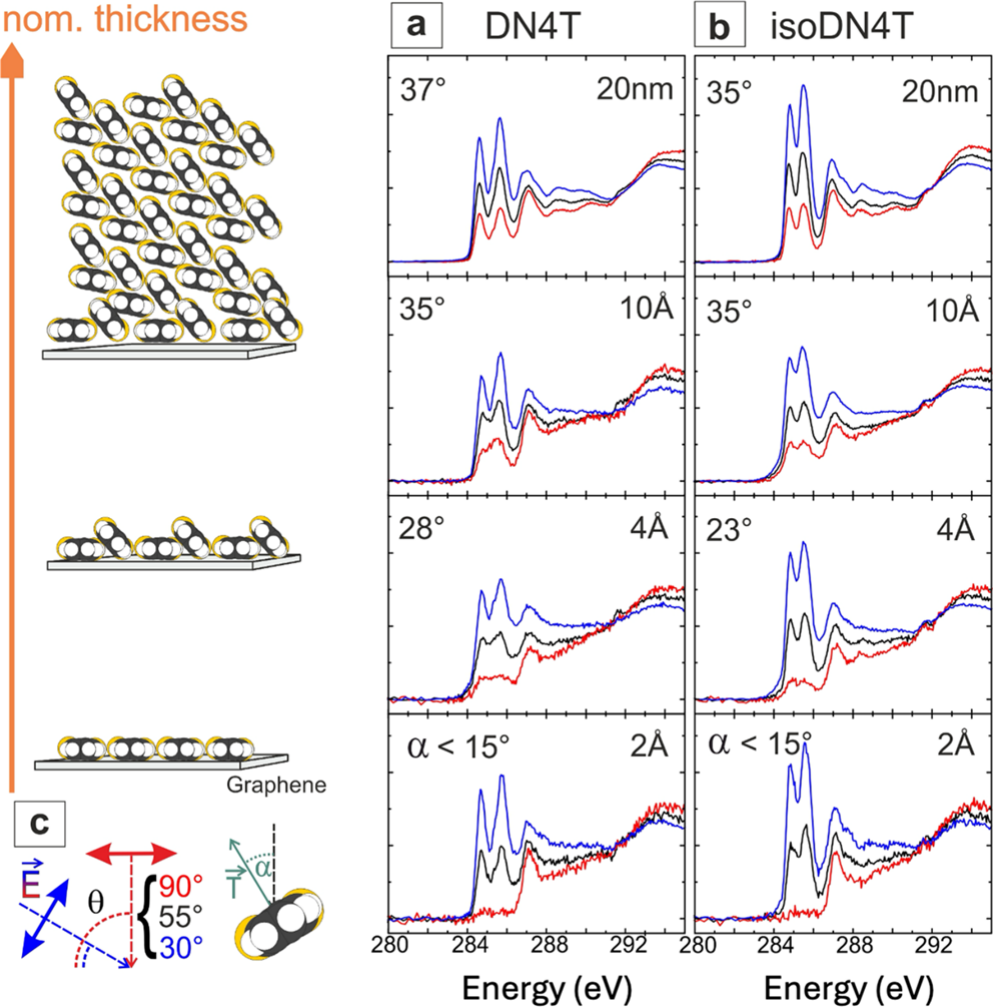
Thickness-dependent NEXAFS spectra for DN4T (a) and isoDN4T (b). The orientation of the molecules deposited on graphene on quartz glass is determined as a function of the electric field vector of the synchrotron light (*E⃗*) and the transition dipole moment vector of the molecule (*T⃗*). The incident angle (θ) is the angle between *E⃗* and the surface normal, the inclination angle (α) is the effective angle between the transition dipole moment vector, represented by a green arrow and the surface normal as depicted in panel (c).

To qualitatively assess the molecular orientation in thin films, we conducted angle-dependent NEXAFS dichroism measurements. Due to the substantial background signal from HOPG we used single graphene layers on quartz glass substrates and applied a background subtraction of the remaining graphene related π* resonance as described in previous work.^49^ These measurements provide insight into the evolution of the molecular orientation relative to the surface normal with increasing coverage.^50^ The NEXAFS spectra, shown in Figure 6a,b, feature distinct π* resonances between 283–288 eV, corresponding to C 1s excitations into unoccupied molecular π* orbitals. At higher photon energies, broader spectral features associated with σ* orbital transitions above the ionization threshold are observed. These NEXAFS signatures show high similarity to previously studied related dinaphthothienothiophene (DNTT).^23^ In NEXAFS, the detected Auger electron yield is maximized when the transition dipole moment vector (*T⃗*), schematically depicted in Figure 6c, aligns with the electric field vector (*E⃗*) of the incident light and minimized when they are perpendicular.^51^ For our flat-lying molecules, minimal intensity is expected when the electric field vector is parallel to the surface. Our results confirm this, indicating molecular inclination angles below 15° for nominal thicknesses of 2 Å, suggesting an essentially flat orientation in submonolayer coverage. At higher nominal thicknesses, the inclination angle increases, saturating at around 37° for DN4T and 35° for isoDN4T, which indicates a transition to inclined molecules beyond the monolayer, presumably facilitating a molecular herringbone/denser packing that was identified for both presently studied compounds,^14^ a structural motif indeed common for such molecules.^20,50,52,53^ The inclination angles derived from NEXAFS measurements contain contributions from all present inclination angles. When deriving inclination angles from NEXAFS measurements, it is important to account for contributions from all molecules within the unit cell. For films with a herringbone packing motif, where molecules align with the long axis parallel to the substrate surface, this results in a weighted average of the individual molecular inclinations. In the case of DNTT, which exhibits a similar herringbone packing motif, NEXAFS dichroism measurements of DNTT films grown on Ag(111) have revealed an average tilt angle of about 33°. This value is quite similar to the present case, suggesting a similar scenario.^23^

From this perspective, the observed HOMO splitting-like feature in isoDN4T is likely a manifestation of intermolecular interactions within a herringbone geometry. Valeev et al. have pointed out that such energy splitting results not merely from intermolecular wave function overlap but also from intermolecular electronic polarization interactions.^26^ These effects emerge due to the nonuniform electrostatic potential around the molecules, where the hydrogenated edges exhibit a positive potential, and aromatic backbones display a comparably negative potential.^26^ In a herringbone geometry, where molecules adopt face-to-edge orientation, intermolecular polarization can become dominant, leading to a large energy difference between the HOMO levels, analogous to a splitting-like situation observed previously for π-stacked lead-phthalocyanine bilayers,^24^ as well as for picene and DNTT dimers.^54^ Considering the small intermolecular transfer integral distribution within isoDN4T single crystals, previously reported to range between 17–33 meV, the observed 430 meV energy split of the HOMO feature in multilayer is more likely due to intermolecular polarization.^14^ The polarization-induced energy difference between the HOMO levels of neighboring molecules creates an electrostatic potential barrier, which can impede charge carrier hopping in certain directions. The magnitude of this effect is strongly influenced by the fine details of molecular packing. Notably, DN4T thin films do not exhibit this behavior, suggesting a substrate-induced phase persisting into the multilayer regime. The distinct behaviors of these two isomeric molecules underscores not only the critical role of molecular structure for the resulting electronic properties but also subtle differences in molecular packing. These differences arise from an interplay of intermolecular and substrate-mediated interactions, which can significantly impact the electronic properties of organic semiconductor thin films. The electrostatic potential landscape at the surface of the substrate along with intermolecular interactions dictate the thin film structure, thereby its transport properties. These aspects, however, have not yet been sufficiently understood and substantial future work is required to arrive at knowledge-guided molecular design for deliberate adjustment of structure and properties of molecular semiconductors.

## Conclusions

We demonstrated that the isomeric DN4T and isoDN4T molecules predominantly assume a nearly flat-on orientation on HOPG at submonolayer coverage, where intermolecular interactions are smaller than those between molecule and substrate. This is unequivocally concluded on from STM, ARUPS, and NEXAFS data. The UPS spectra distinctly show the electronic valence structure, particularly evident through the clearly defined HOMO peaks at the lowest binding energy sides of the spectra. The analysis of the vibrational progression observed in the HOMO peaks enabled us to estimate the molecular reorganization energies and polaron binding energies for both DN4T (λ = 98 meV, *E*_pol_ = 49 meV) and isoDN4T (λ = 168 meV, *E*_pol_ = 84 meV). These experimentally derived values are consistent with those predicted theoretically and correlate well with the hole mobilities measured in organic field-effect transistors. This supports the hypothesis that charge transport is more efficient in DN4T than in isoDN4T. Additionally, our thickness-dependent UPS measurements revealed a notable splitting-like feature of approximately 450 meV of the HOMO level of isoDN4T. While such a phenomenon might initially be attributed to wave function overlap, our discussion favored intermolecular electronic polarization as the main contributor. This finding might provide an explanation for the significantly reduced experimental hole mobility observed in isoDN4T, a discrepancy not fully addressed by the existing theoretical results. To further investigate the underlying mechanism of the HOMO splitting-like feature, acquiring additional structural data on the initial layers is necessary. Conducting analogous measurements on alternative substrates could shed light on substrate-induced polymorphism, enriching our comprehension of the complex interplay between molecular structure and electronic properties. Gaining such insights is crucial for the strategic engineering of thin film structures, which could pave the way for improved performance in devices based on organic semiconductors. This knowledge is essential for devising strategies to engineer thin film structures, potentially leading to enhanced performance in organic semiconductor devices.

## Data Availability

The data underlying this study are available in the published article and its Supporting Information.

## Supporting Information Available

Additional XPS spectra of the S 2p core level for DN4T and isoDN4T films; scaling factor vs emission angle geometry for correcting HOMO intensity; deconvolution of angle-resolved UPS spectra of DN4T submonolayers; comparative plot of reorganization energies of DN4T and isoDN4T as a function of emission angle; thickness-dependent UPS spectra of the secondary electron cutoff region for DN4T and isoDN4T (PDF)
