# Supplementary material for: Two Isomeric Thienoacenes in Thin Films: Unveiling the Influence of Molecular Structure and Intermolecular Packing on Electronic Properties
Source: J Phys Chem C Nanomater Interfaces. 2024 Dec 2;128(49):21228–36. doi: 10.1021/acs.jpcc.4c06741 (PMC11648078; doi:10.1021/acs.jpcc.4c06741)
Supplement: Supplementary file 1 — jp4c06741_si_001.pdf [file jp4c06741_si_001.pdf]

Supporting Information
for

Two Isomeric Thienoacenes in Thin Films: Unveiling the Influence of Molecular Structure and Intermolecular Packing on Electronic Properties

Christos Gatsios[1], Maximilian Dreher[2], Patrick Amsalem[1], Andreas Opitz[1], Remy Jouclas[3], Yves Geerts[3,4], Gregor Witte[2], and Norbert Koch[1,5*]

[1]Institut für Physik & Center for the Science of Materials Berlin (CSMB), Humboldt-Universität zu Berlin, 12489 Berlin, Germany

[2]Department of Physics, Philipps-Universität Marburg, 35037 Marburg, Germany

[3]Laboratoire de Chimie des Polymères, Faculté des Sciences, Université Libre de Bruxelles (ULB), Boulevard du Triomphe, CP 206/01, Bruxelles 1050, Belgium

[4]International Solvay Institutes for Physics and Chemistry, Université Libre de Bruxelles (ULB), Boulevard du Triomphe, CP 231, Bruxelles 1050, Belgium

[5]Helmholtz-Zentrum Berlin für Materialien und Energie GmbH, 12489 Berlin, Germany

*E-Mail: norbert.koch@physik.hu-berlin.de

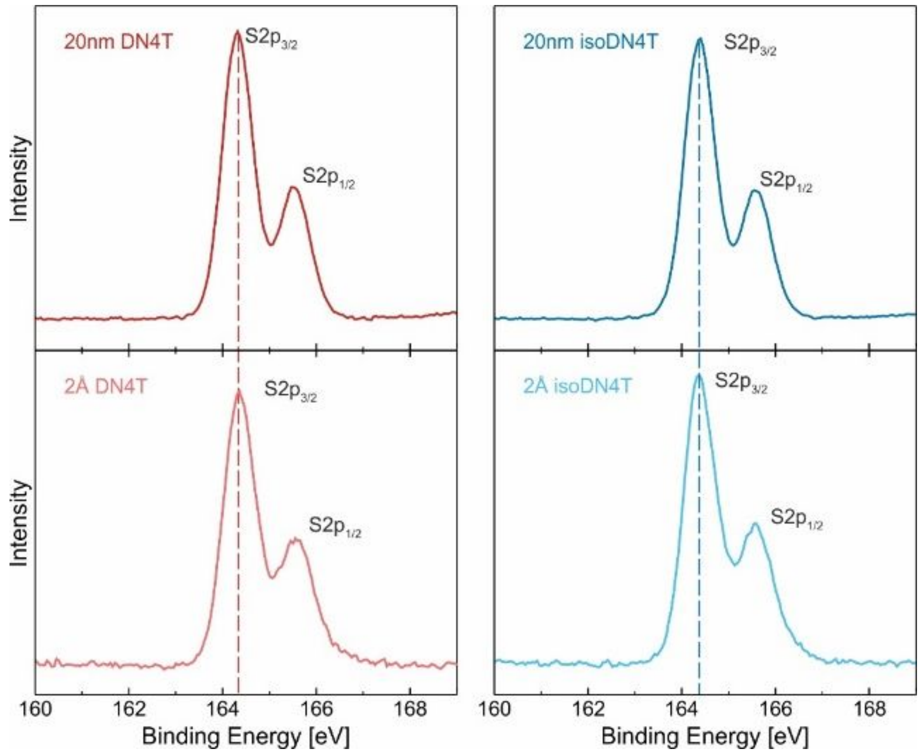

Supplementary Figure S1: Additional XPS spectra of the S 2p core level for DN4T and isoDN4T films with submonolayer (2 Å) and multilayer (20 nm) coverage on HOPG. Spectra were recorded with an excitation energy of 350 eV. The S $2p_{3}/_{2}$ core level peaks at submonolayer coverage align with those observed in similar molecules, such as DNTT ((Breuer et al., ACS Appl. Mater. Interfaces 2017, 9, 8384, DOI: 10.1021/acsami.6b15902)), indicating weak interactions with HOPG, with sulfur atoms remaining within the aromatic framework rather than forming covalent bonds. Furthermore, the lack of significant spectral changes between submonolayer and multilayer coverage suggests that any intermolecular interactions in the multilayers do not impact the core electronic levels.

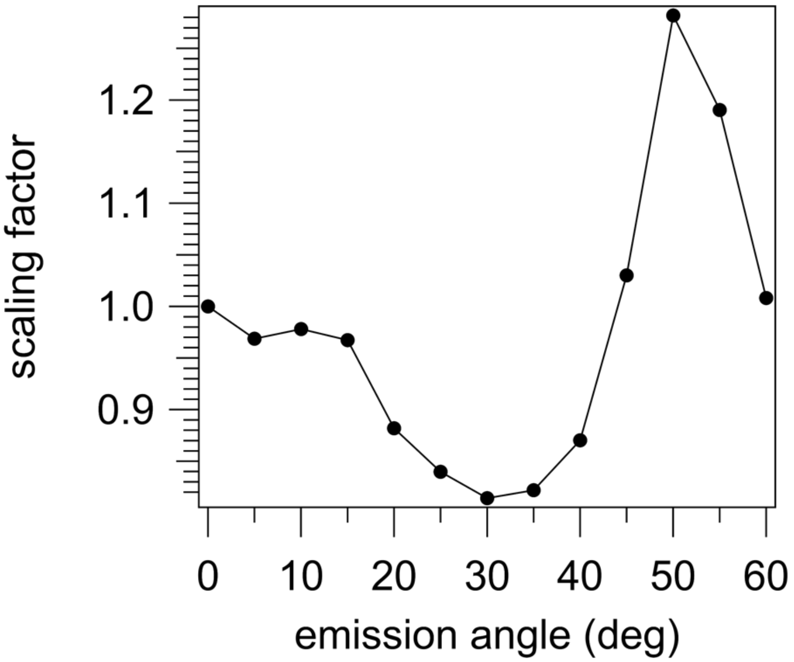

Supplementary Figure S2: Additional experimental details. Scaling factor vs. emission angle geometry. This graph is obtained after measuring the Fermi edge of a polycrystalline silver substrate for each emission angle geometry. To obtain a scaling factor the intensity was normalized with respect to the intensity of the Fermi edge at normal emission angle. The scaling factors were used to apply a correction to the HOMO intensity of DN4T and isoDN4T.

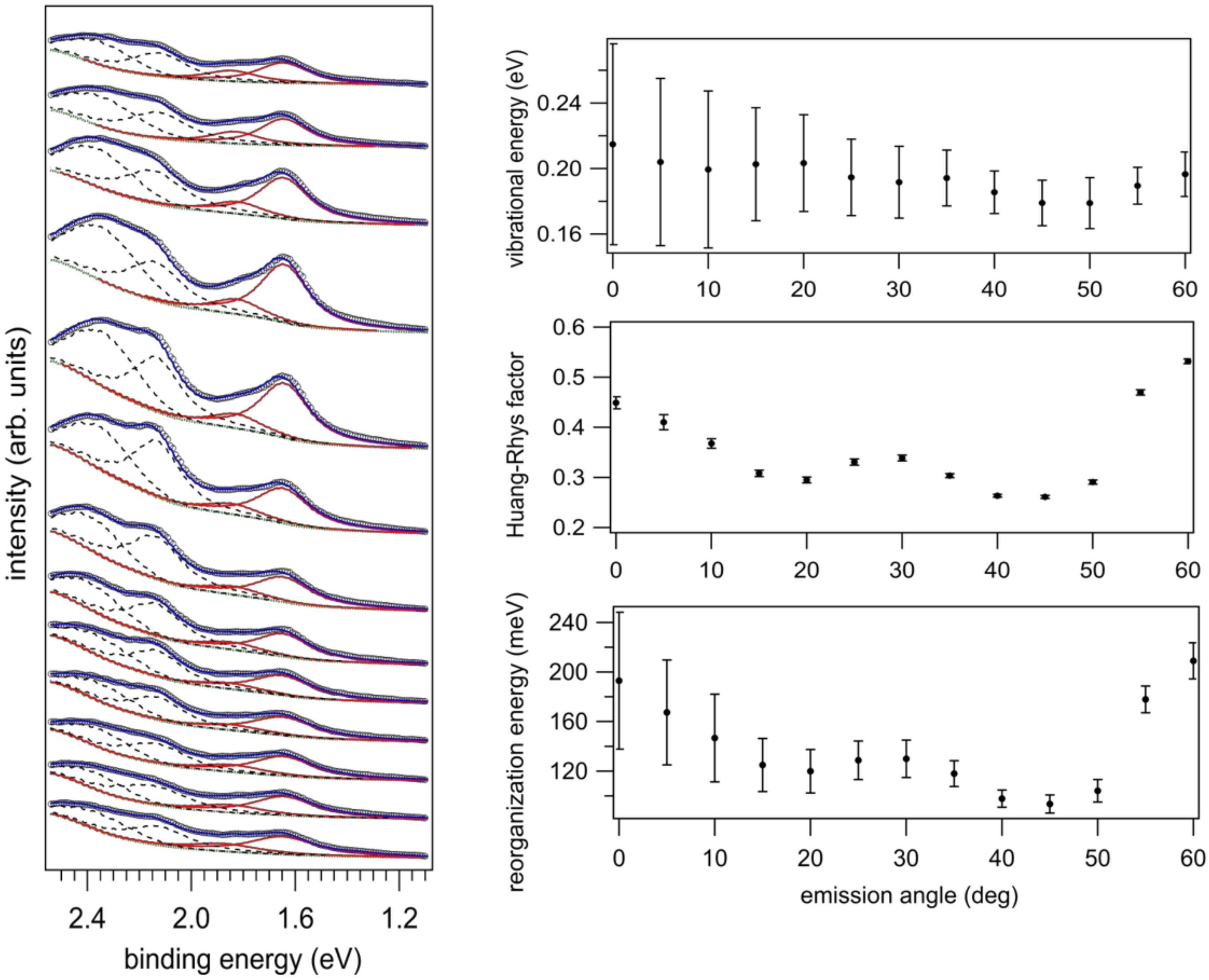

Supplementary Figure S3: Additional details on the data analysis. Deconvolution of angle-resolved UPS spectra of DN4T sub-monolayers. As mentioned in the main text the HOMO was deconvoluted by two Voigt functions which are depicted by the red curves. The black dashed lines show electronic peaks corresponding to deeper energy levels. The empty black circles are the raw data, and the blue solid line shows the fitted curve. The background intensity which is subtracted by each spectrum is depicted by dashed green curve. The results of least-squares fitting, namely vibrational energy, Huang-Rhys factor and reorganization energy are graphically shown as a function of the emission angle. The error bars correspond to the statistical error arising from the fitting analysis.

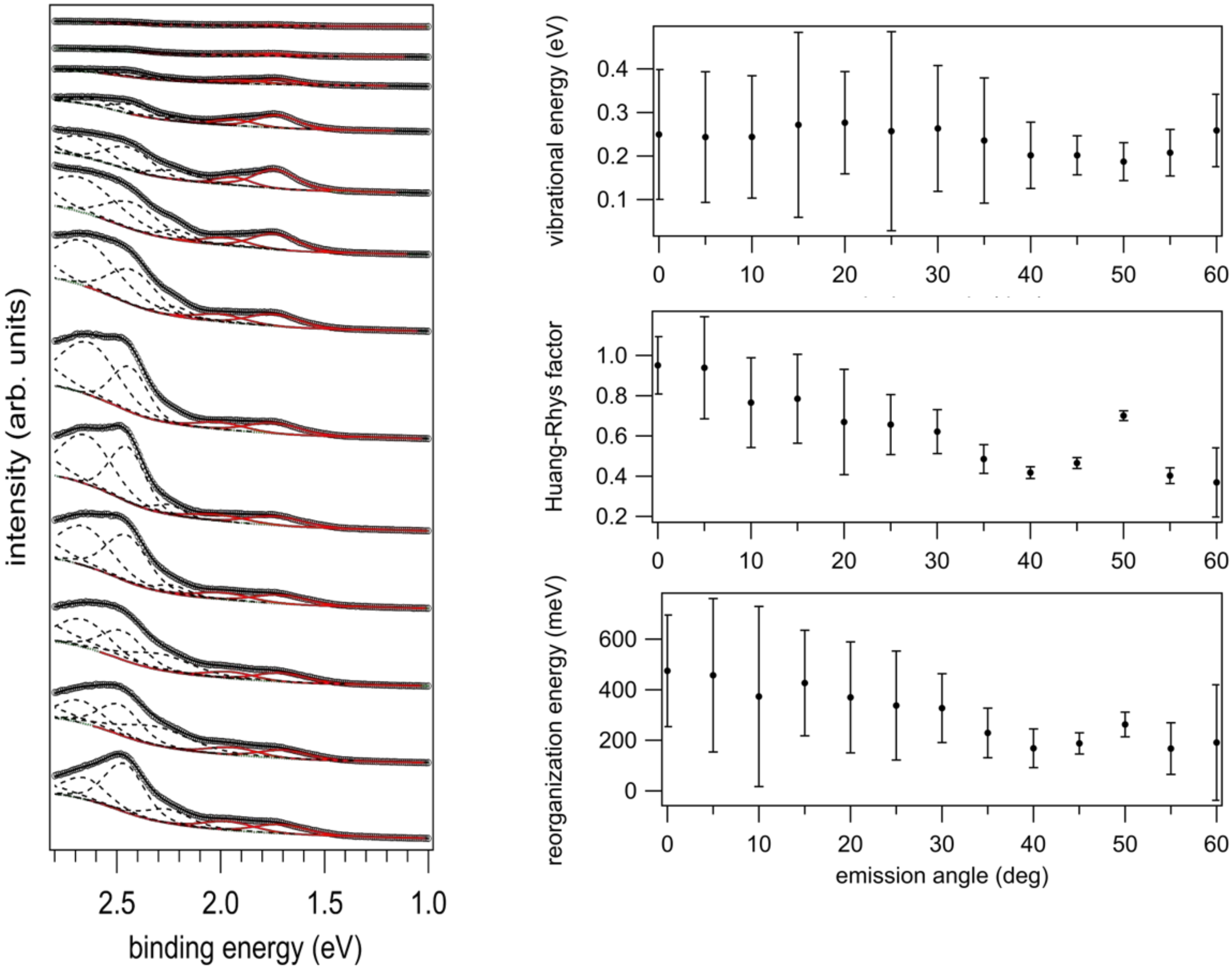

Supplementary Figure S4: Additional details on the data analysis. Deconvolution of angle-resolved UPS spectra of DN4T sub-monolayers. As mentioned in the main text the HOMO was deconvoluted by two Voigt functions which are depicted by the red curves. The black dashed lines show electronic peaks corresponding to deeper energy levels. The empty black circles are the raw data and the blue solid line shows the fitted curve. The background intensity which is subtracted by each spectrum is depicted by dashed green curve. The results of least-squares fitting, namely vibrational energy, Huang-Rhys factor and reorganization energy are graphically shown as a function of the emission angle. The error bars correspond to the statistical error arising from the fitting analysis.

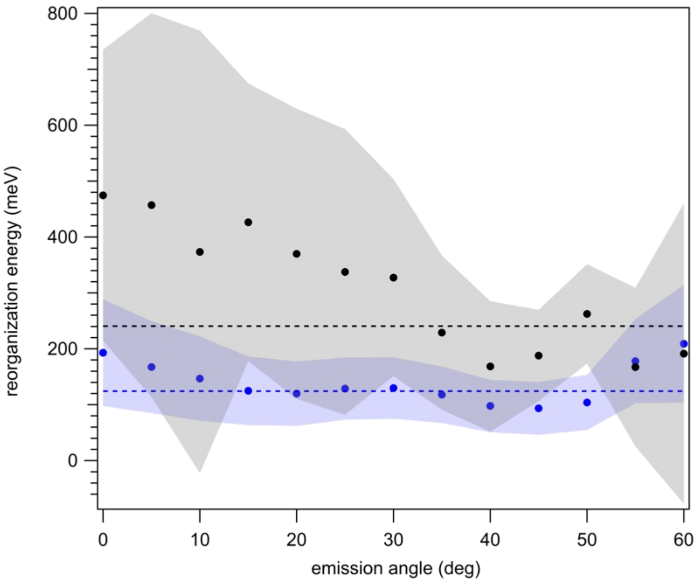

Supplementary Figure S5: Additional details on the data analysis. Comparative plot of the reorganization energies of DN4T and isoDN4T as a function of emission angle. The solid blue points represent DN4T, while the black points correspond to isoDN4T. The shaded grey areas indicate the total uncertainty, including both statistical and experimental errors (80 meV). Dashed lines show a linear fit, representing the weighted average of the data points. On average, the reorganization energy of DN4T is lower than that of isoDN4T.

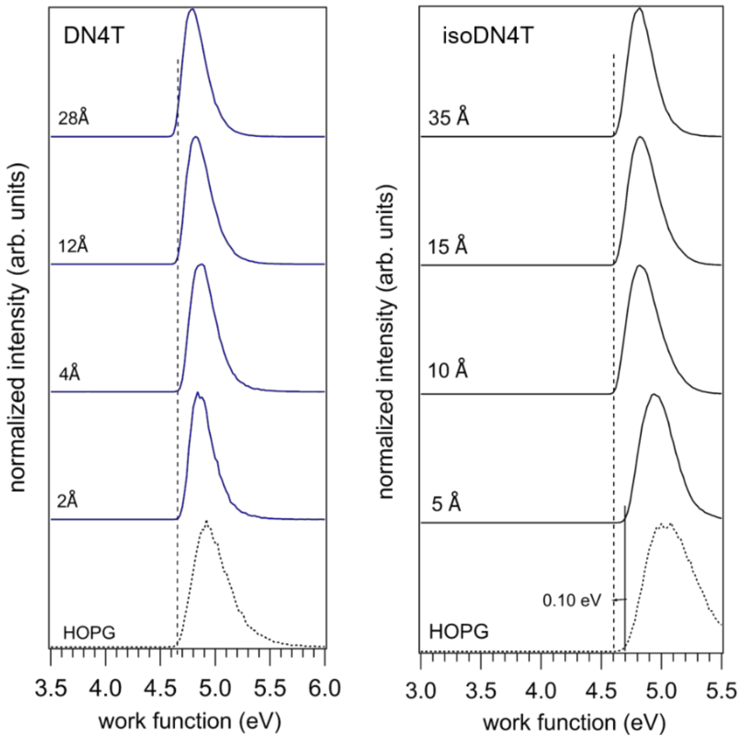

Supplementary Figure S6: Additional experimental details. Thickness-dependent UPS spectra of the secondary electron cutoff region (SECO) of DN4T and isoDN4T. The onset of the SECO is a measure of the work function and is determined from the intersection between the linear extrapolations of the flat background and the onset curve. Increasing the nominal thickness there is no shift of the SECO in isoDN4T, suggesting no change in the charge distribution at the surface or the presence of surface dipoles. In isoDN4T there is a small shift of the SECO between 5 and 10 Å which might be related to the HOMO splitting-like feature observed at the same deposition step. The formation of a layer of molecular dimers could change the charge distribution at the surface resulting in such a shift. Alternatively, this small shift might be linked to different charging compensation in higher thicknesses compared to the sub-monolayer case. A small positive charge at the surface would shift the SECO towards lower work functions.
